# Understanding the (inter)disciplinary and institutional diversity of citizen science: A survey of current practice in Germany and Austria

**DOI:** 10.1371/journal.pone.0178778

**Published:** 2017-06-27

**Authors:** Lisa Pettibone, Katrin Vohland, David Ziegler

**Affiliations:** 1 Public Engagement with Science, Museum für Naturkunde Berlin, Berlin, Germany; 2 Rachel Carson Center for Environment and Society, Ludwig-Maximilians-Universität Munich, Munich, Germany; VU University Amsterdam, NETHERLANDS

## Abstract

Citizen science has become more popular in recent years, quickly taking on a variety of potentially conflicting characteristics: a way to collect massive data sets at relatively low cost, a way to break science out of the ivory tower and better engage the public, an approach to educate lay people in scientific methods. But the extent of current citizen science practice—the types of actors and scientific disciplines who take part—is still poorly understood. This article builds on recent surveys of citizen science in PLOS One by analyzing citizen science practice in Germany and Austria through the projects on two online platforms. We find evidence supporting previous findings that citizen science is a phenomenon strongest in biodiversity and environmental monitoring research, but at home in a number of scientific fields, such as history and geography. In addition, our survey method yields new insights into citizen science projects initiated by non-scientific actors. We close by discussing additional methodological considerations in attempting to present a cross-disciplinary overview of citizen science.

## Introduction

Citizen science is increasing in popularity, in the media, in scientific circles (as seen in recent surveys in PLOS One [1,2]), and with policymakers. Proponents argue that citizen science does not have disciplinary boundaries, and is therefore an approach to include the public in all fields of scientific research [3]. As the field begins to jump disciplinary boundaries—with field ecologists sharing best practice with astronomers and practitioners in different fields joining citizen science networks and developing political strategies [4]—it is important to test this claim. We attempt to do so by presenting a survey of current citizen science practice in Germany and Austria, measured by projects on two online citizen science platforms. In doing so, we ask the question: *How—by whom and in what fields—is citizen science practiced today*?

The term “citizen science” originated in an Anglo-American context and has become more clearly defined there over the past two decades. It signifies a variety of activities, such as in conservation biology and environmental monitoring [5,6], serious games in the natural sciences [7–9], activist activities that use research to influence policy [10], and even research by government agencies [11]. Within the citizen science community, subgroups have begun to emerge that define themselves more precisely with respect to their participatory model, such as “crowd science” (in which large data sets are created by faceless volunteers) [12,13] and “extreme citizen science” (which attempts to include citizens in all aspects of the research process) [14].

What is citizen science? Definitions have multiplied in the past few years that emphasize different aspects or practices of “public participation in scientific research,” an umbrella term used by Shirk et al. (2012) [15]. They look at how members of the public interact with professional scientists; in citizen science, volunteers’ contribution is generally merely to contribute data, although they may collaborate with scientists on project design, data analysis, and dissemination of results in specific cases [15]. “Co-creation” of process and results falls to other participatory research forms [15]. Wiggins and Crowston (2011) also understand citizen science as “a project in which a professional researcher collaborates with volunteers in scientific research” [12, p. 3]. Other definitions sometimes include additional features, such as a contribution to education [16,17], conservation [18,19], or democracy [20,21], or projects’ openness to potential contributors and availability of data inputs [13]. Researchers have also developed typologies of certain aspects of citizen science, such as forms of participation [22–24] or discussed recent developments and potential directions for citizen science more generally [11,18,25]. The European Citizen Science Assocation describes citizen science through 10 principles of good practice, such as active involvement of citizens, scientific outcomes, open and transparent processes, and benefits for all participants [26].

Some authors describe citizen science projects initiated by societal [10,27] and other actors [11], which have additional objectives to advancing scientific knowledge. Instead, projects coordinated by non-scientific actors are interested in policy change [10,11], education and knowledge transfer [28], sharing scientific knowledge informally or in small publications focusing on narrow scientific subjects (e.g., presentation of a relevant new insect sighting during a bioblitz in a local entomological journal [29]), or simply see scientific inquiry as an engaging hobby [30]. These studies reveal a more complicated role for “citizens” than simply as volunteers in scientific research.

To date, few comprehensive surveys of citizen science exist, although the breadth of such research is growing. Wiggins and Crowston (2011) distinguish between different project structures and goals, such as conservation or online activities [12]. Scheliga et al. (2016), use the term crowd science to describe projects from the German citizen science platform buergerschaffenwissen.de (which frame themselves as citizen science), find key project objectives to range from the generation of scientific knowledge to broader interest in a topic [31]. In none of these definitions, even more those interested in the role of “citizens,” are actors understood more precisely than as “scientists” and non-scientists [32]. A better understanding of who actually participates in citizen science projects can help shed light on the roles of citizens and scientists and project goals.

In addition, few studies have attempted to survey citizen science practice across all disciplines or fields of practice. Some recent work has sought to summarize practice within a single field or discipline, such as ecology [33,34], astronomy [22], and health research [23,24], focusing on key features and challenges within the specific research domain. Recent articles by Follett and Strezev (2015) and Kullenberg and Kasperowski (2016) in PLOS One appear to be the first to attempt to survey the whole citizen science landscape [1,2]. Both analyze Web of Science (and SCOPUS, in the former) publications to reveal keywords and disciplinary or thematic focal points of citizen science in the academic literature. Both papers find dramatic growth in publications from an initial date around 1997. Follett and Strezev (2015) find the main thematic focus in conservation biology and environmental monitoring, along with additional strands in astronomy, health, and environment, which tracks with Kullenberg and Kasperowski’s three clusters in citizen science (again, primarily in conservation biology and the natural sciences), distributed geographic data, and public engagement, the latter with strong links to social science discussions [1,2]. Both articles admit an important limitation to their review: their literature-based analysis excludes citizen science efforts not represented in Web of Science (or SCOPUS), such as digital humanities and projects with a non-publication focus [1,2]. Although the English-language literature primarily describes citizen science as taking place in the natural sciences, some efforts have emerged in the social sciences, such as urban planning or action research [14]. Social science scholars and practitioners of approaches such as participatory or action research used the term “citizen science” sporadically in the 1990s and early 2000s [10,21], as well as link such approaches to more theoretical discussions of science–society interactions [21]. The social sciences appear to have found more resonance with other terminology [35,36]. Very recently, some scholars have sought to re-introduce their practice into citizen science with “citizen social science” [37] or calls for more citizen science efforts in the social sciences, despite critical differences such as normativity or ethical concerns in research involving human behavior [38]. It appears that few direct links to the humanities exist in academic journals [1,2], but this may be due at least in part to different publication practices (see below). In Germany, historians at the University of Erfurt are building a network of practice among citizen science activities in the humanities [39]. These efforts indicate that citizen science is practiced across the disciplinary spectrum. It is time to draw a better empirical picture of which disciplines are well represented, using methods that can capture disciplines with different publication practices.This article seeks to address these gaps—the roles of actors and the disciplines practiced in citizen science—by focusing on analysis of current citizen science projects. It does so by examining current practice in Germany and Austria, two countries of interest because of their strong histories of lay person–led “popular science” (*Populärwissenschaften*, [40]) as well as the recent surge of citizen science practice. In doing so, we expand upon previous research in two ways. First, we analyze the role of different types of actors in citizen science projects to expand on previous surveys. Here we ask: *What types of actors initiate and coordinate citizen science projects*? If it is true that some citizen science projects are absent from the scientific literature, for example because they focus on other goals, we should see actors beyond scientific institutions initiating and coordinating projects. Second, we analyze the disciplinary make-up of the current citizen science landscape in Germany, in order to determine, *In what scientific fields is citizen science practiced*? This will help us determine whether citizen science truly has cross-disciplinary character, or indeed has disciplinary boundaries.

### Defining citizen science

Due to the diversity and heterogeneity of citizen science approaches, it is both important to lay out some generally accepted characteristics for citizen science and impossible to find a standard definition. As shown in the introduction, definitions generally agree that citizen science is the pursuit of scientific knowledge that includes some sort of active participation by non-scientists. We also use these as two main criteria that all approaches have in common:

- **scientific inquiry.** Citizen science seeks to advance scientific understanding, by using scientific methods to generate or analyze data, test hypotheses, or answer questions. Citizen science goes beyond activities that use non-scientific methods (such as public engagement or volunteering) or simply repeat experiments to advance scientific literacy without developing new knowledge (as in many educational activities).
- **inclusion of non-scientific actors.** Although the “citizens” in citizen science can be virtually anyone, they should include people who are not professionally engaged in scientific research, either as paid scientists or those pursuing further qualification in a scientific field (e.g., doctoral students). Generally, citizen science projects should be open to the public in some way, engage volunteers actively, and offer benefits for participation.

Beyond this, different authors focus on key aspects of citizen science, such as project characteristics, objectives, involvement of volunteers, or additional benefits. Missing are the types of actors involved and the disciplines that use citizen science approaches. Indeed, the purpose of this paper is to investigate these two issues that have been less well addressed in the literature.

## Methods

We use a two-part approach to survey current citizen science practice in Germany and Austria: a quantitative analysis of 97 citizen science projects on citizen science platforms in Germany and Austria, supplemented by qualitative findings from GEWISS (described further in the discussion section). The authors of this paper worked on or with GEWISS, as project coordinator, lead, and editor of the companion web platform buergerschaffenwissen.de, respectively [41].

**Fig 1:**
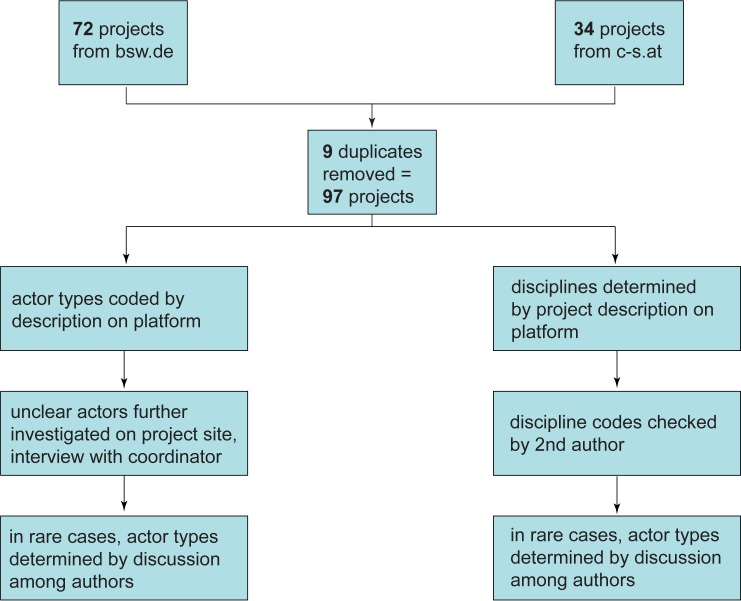
Overview of quantitative analysis process. Data set included in S1 Appendix.

Survey data come from two major German-language citizen science platforms: the German citizen science platform buergerschaffenwissen.de (BSW), which had 72 projects online in August 2016, as well as the Austrian citizen science platform “Austria researches” (*Österreich forscht* or citizen-science.at), which was home to 34 projects at that time. Nine projects were featured on both platforms and counted only once, resulting in a total of 97 unique citizen science projects (see Fig 1). Information on both platforms is provided by the projects themselves, who provide a project title, description, list of participating institutions, and additional information such as contact person, project website, and photos. The two data sets can thus be considered of comparable quality. Survey data were collected in August 2016 by collating project descriptions from the individual project pages on both sites.

Data were analyzed based on projects’ own descriptions on the platforms. We chose to analyze actor types as a proxy for purpose. We assumed that non-scientific institutions, such as nonprofit groups or government agencies, would be less likely to publish in academic journals. Therefore the relative importance of these actors might suggest how much of current citizen science practice might be omitted by surveys of the academic literature. Actors were determined based on the actors listed under “institutions.” Organizations that did not clearly fit actor types were further researched on the project home pages and, if necessary, by direct contact with project coordinators.

Scientific disciplines were extrapolated based on our best guess as to what type of scientific journal the project would theoretically submit its research, based on project descriptions provided on the platform. This approach was used to capture potential scientific disciplines as represented in (or omitted from) Web of Science or SCOPUS and, like actor types, to get a sense of how well the citizen science community may be represented by those methods.

Both platforms attempt to be cross-disciplinary repositories of citizen science activities. Citizen science projects on buergerschaffenwissen.de were recruited through GEWISS events, workshops and presentations on citizen science at various events from 2014 to 2016, as well as word-of-mouth. Projects on *Österreich forscht* were recruited through similar means, including two conferences on citizen science in Austria (Dörler & Heigl, personal communication, 2016). The projects on these platforms represent a broad cross-section of current citizen science practice in German-speaking countries, including projects not necessarily focused on scientific publication. Due to our expertise in the German and Austrian citizen science community, we did not analyze platforms in other countries, such as Switzerland or Belgium, or international platforms such as SciStarter and Zooniverse. Instead we used our experience of the citizen science community developed during the GEWISS project, which allowed us to compare survey findings from the platform to discussions at GEWISS events and better understand how representative these platforms are of the German-speaking community. Such a qualitative check on the data would not have been possible with platforms in other countries.

This paper seeks to test the finding of Kullenberg and Kasperowski (2016) and Follett and Strezov (2015) by supplementing the methods used in those surveys [1,2]. Still, these data are constrained by several factors: 1) the project’s interest in joining, 2) adherence to criteria for good citizen science practice (see definition above), 3) active status that accepts potential participants, and 4) a project website. However, it appears that these constraints posed only a minor hurdle, as seen by the project submissions rejected by buergerschaffenwissen.de. Of the 9 projects rejected by platform editors, 5 failed to meet scientific standards (i.e., pseudoscience); 2 included public participation without a significant scientific component (e.g., surveys); 1 was deemed premature for the platform (the associated app was not complete); and 1 was organized by a group with significant commercial activities too closely linked to the project. The platform projects are analyzed in the following results section. In addition to the quantitative analysis there, additional qualitative findings based on GEWISS research (in the form of workshop organization, contacts with additional activities not on the two platforms, and participant-observation at external events) inform the subsequent discussion.

## Results

### Key actors in citizen science

**Fig 2:**
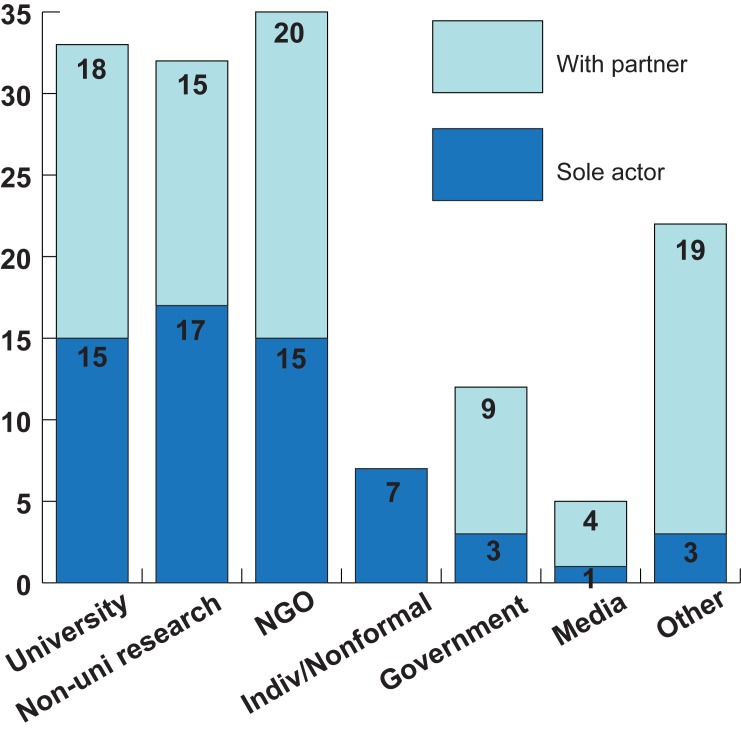
Engagement in platform projects, by actor type. N = 97, from data recorded from platforms 2016-08-15. Actor types collected from project descriptions on platforms, project websites, and interviews with project coordinators (S1 Appendix, project analysis).

The citizen science projects on the platform are coordinated by a diverse range of organizational types and reveal diverse motivations to engage in citizen science. Key actors here are grouped into scientific institutions, society-based groups, and other actors (government agencies, the media, and others), which are further subdivided in the graphs (Fig 2).

Scientific institutions represent actors based in professional research, which we assume would seek to produce findings that could be published in the scientific literature. This group includes both *universities* and *non-university research institutes*. Non-university research institutes include members of professional associations such as the Leibniz or Helmholtz Associations as well as loose-knit scientific infrastructure networks such as BOINC and GBIF, which have members from various research organizations in a specific scientific field.

Society-based groups vary widely and are distinguished from scientific institutions in their interest in societal change. These groups include *non-profit organizations* (i.e., NGOs) focused on political or social issue engagement (such as environmental groups), some of which have professional research components (such as BUND or NABU) or are structured as research organizations (such as UfU). In addition, we consider *independent groups* interested in scientific research outside the academic context (*Fachgesellschaften* in German), which we group with engaged individuals and small groups of individuals.

Finally, government agencies and media organizations were involved in several projects, earning further actor categories. This classification scheme allowed us to determine the potential range of citizen science activities not captured by previous surveys, specifically those projects initiated by non-scientific actors.

Fig 2 shows the number of projects organized by different actors, either as sole coordinators or in cooperation with others. Projects coordinated by multiple actors of the same type (e.g., by two universities or several individuals) were categorized as having a single actor of that type. Therefore, 33 of the 97 projects (34%) on both platforms include engagement by a university. The sub-classification as sole actor or with partners allows us to suggest the centrality of different actor types. For example, although government agencies are engaged in 12 projects, they are the sole coordinator of only 3, indicating their likely role as a supporting player in citizen science.

**Fig 3:**
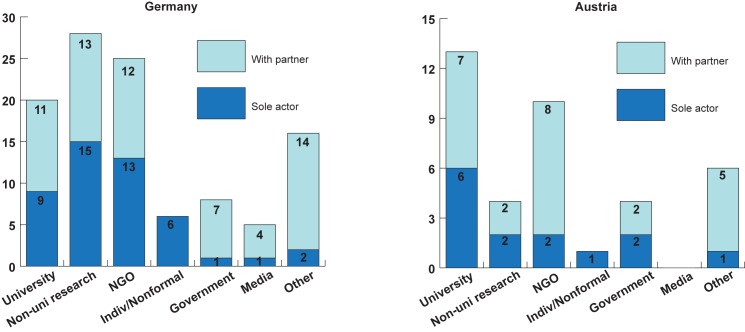
Engagement in platform projects by actor type, country comparison. N = 97, from data recorded from platforms 2016-08-15. Country reflects country the project takes place in, not necessarily the platform on which it is listed, as listed in the project description (S1 Appendix, project analysis).

Fig 2 highlights the diversity of actors engaged in citizen science. As expected, scientific institutions are a leading actor, involved in 65 projects (67%). Here, research institutes outside the university system—primarily members of research associations such as Leibniz and Helmholtz—represent the largest number of projects in Germany, whereas universities are more important scientific players in Austria (see Fig 3).

In addition, a large number of projects are coordinated by society-based organizations or individuals. 22 of these projects (24% of the total) are coordinated by a single NGO or individual. Of the 20 projects with partners (21%), 10 include partnership with scientific actors. The other 10 are difficult to generalize and include a mix of partnerships with government, media, and other actors. Finally, a full 22 projects include engagement by other actors (not including government and media), 3 alone and 19 in partnership. In total, 41% of projects on the two platforms do not include a scientific partner (42% in Austria and 40% in Germany).

### Scientific disciplines

**Fig 4:**
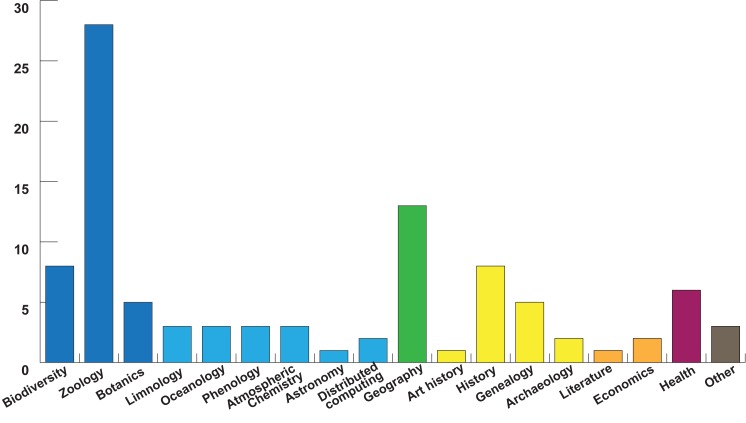
Disciplinary focus of platform projects. N = 97, from data recorded 2016-08-15. Disciplinary categories imputed by authors from project descriptions (S1 Appendix, disciplines).

Scientific disciplines were determined by project descriptions provided by projects themselves on both platforms and project websites. We coded each project according to a single research discipline that reflects the discipline research findings might be published under, were a scientific article to be written. In limited cases, multiple disciplines were possible (such as an online platform to provide distributing computing for numerous projects in the natural sciences). Here, we discussed and recorded the mostly likely discipline for publication (in the above case, computing). The categories here are thus illustrative of disciplines that could benefit from citizen science activities on the two platforms. Disciplines are represented in Fig 4.

**Fig 5:**
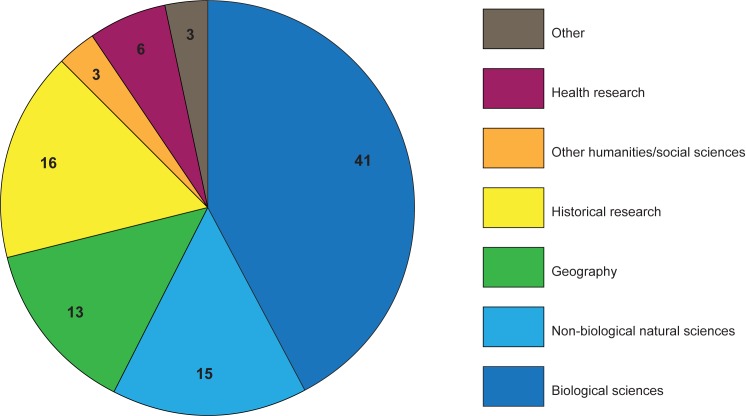
Disciplinary clusters. N = 97, from data recorded 2016-08-15. Disciplinary clusters created by authors (S1 Appendix, disciplines).

The 97 projects on both platforms support previous findings that biological research (here including biodiversity, zoology, and botanics) is the most well represented discipline (Follett & Strezov have it representing over 70% of publications, [1]), with additional activities in geography, astronomy, and health research [1,2]. The disciplinary analysis also reveals surprisingly strong representation in the humanities and social sciences. Although biology is a popular discipline among platform projects, a number of additional disciplines have at least one project, with atmospheric chemistry, archaeology, history, genealogy, geography, and health as additional disciplines with at least 3 projects (see Fig 5 for an overview). The relative weight of biological projects, at 42%, has a strong plurality but no absolute majority (see Fig 5). We also found slightly fewer projects in the non-biology natural sciences (15%) than expected.

In addition, we found a surprising cluster in historical research—including local or regional history, genealogy, art and cultural history, and archaeology—with 16% of the total. These categories were not shown in either Kullenberg or Follett [1,2], and highlight an important area of citizen science practice not yet seen in the survey literature. Both previous studies themselves acknowledge the potential shortcoming of surveys based exclusively on Web of Science (and SCOPUS) publications in excluding projects not published in such venues. Our findings support these authors’ concerns and suggest that a broader survey that included scholarly publications in the humanities might yield additional results.

## Discussion

In this article, we found that current citizen science practice in Germany and Austria is more diverse than the scientific literature to date has acknowledged, including both a wider range of and more diverse roles for actors than has been captured and a larger number of scientific disciplines than generally discussed. In this section, we further examine both the actors involved in these projects and the disciplines in which citizen science is currently practiced in Germany and Austria.

To do so, we use the survey data as well as our experiences as researchers in GEWISS. As project coordinator and leader, respectively, we engaged in significant network-building activities, presenting the platform in a variety of settings, thus increasing the visibility of citizen science in numerous disciplines and giving us insights into current practice in various contexts [41]. In addition to organizing GEWISS events on aspects of citizen science [4], including “Citizen science beyond the natural sciences” [42] and a follow-up on “Citizen science in cultural studies and humanities” [43] focused on discussed current practice in these areas, we attended events held by numerous disciplines, from environmental protection [44] to digital citizen science [45], transdisciplinary research to science–society interaction [46]. This allowed us to better understand not only whether citizen science is practiced in these areas, but also the relationship between its approach and other attempts to include non-academic actors in scientific research. We would like to share some of our insights from these efforts in order to discuss the presence and absence of certain actors and disciplines in current citizen science practice.

### The diversity of actor types

We demonstrated above the diversity of actors engaged in citizen science. In particular, we found that societal actors—in the form of non-profit advocacy groups, independent scientific organizations, and engaged individuals—play a large role in initiating and coordinating citizen science projects in Germany and Austria. We found that a full 40% of projects are coordinated by such actors. This high number of society-based activities strengthens the argument that scientific literature–based survey methods likely miss a significant portion of citizen science practice, because such projects are less likely to publish in Web of Science journals. It also suggests that more theoretical work is needed to understand citizen science participants beyond the categories of “scientist” and “volunteer.” Indeed, the large engagement of a wide range of non-scientific institutions seems to indicate a more complex and multi-faceted “citizen scientist” than the image of the eager layperson. Scientists as well seem to play roles beyond project initiator in several cases and may be employed by institutions beyond those dedicated primarily to research.

The large number of projects conducted without the involvement of scientific institutions suggests that surveys based entirely on scientific publications cannot capture the full diversity of citizen science practice. Our platform-based survey methodology appears better at capturing the extent of such projects and also supports discussions elsewhere that citizen science projects have goals beyond expanding scientific knowledge. More work is needed to deepen our understanding of the roles played by “citizens” in citizen science.

However, analysis by actor type may deliver an artificially stark portrait of citizen science activities. In fact, not all projects initiated by scientific institutions necessarily have scientific inquiry as their top priority. For example, Expedition Münsterland is listed as a project coordinated by the Westfälische Wilhelms-Universität, suggesting a scientific motivation or peer-review publications as a potential outcome. However, the project is in reality an infrastructure for a range of projects related to the Münster region, which can be initiated by project coordinators, university staff, or the interested public [28,47]. Expedition Münsterland is coordinated by the university’s knowledge transfer office, indicating a stronger focus in science communication and dialogue. The projects undertaken by this initiative often focus on local history [47], but their interest is primarily to build new connections between the university and the surrounding area.

In addition, projects led by societal actors pursue a range of goals. The membership of many independent scientific groups (*Fachgesellschaften*) is composed of enthusiastic researchers whose decades of experience in a specific field—such as entomology—more than makes up for a lack of advanced scientific qualification. Expert citizen scientists may co-write scientific articles together with professional scientists at museums or research institutes (e.g., [48]), and even serve as doctoral advisors. Here citizen science takes the form of lifelong avocation, rather than discrete project, adding another challenge for researchers seeking to capture such activities. Additional work needs to be done to understand these roles and better incorporate them into theoretical understandings of citizen science. Empirical social science research may help illuminate the complex picture of actors’ roles that can only be hinted at here.

Another important actor in numerous citizen science projects was that of scientific infrastructure networks, such as the Global Biodiversity Information Facility (GBIF) or the Berkeley Open Infrastructure for Network Computing (BOINC). These organizations are often initiated and funded at least in part by scientific institutions and focus on building data infrastructure for citizen science. GBIF itself does not necessarily ask research questions of the data it collects, but focuses on building data sets and establishing quality standards so that others can conduct research using their repository. Others have noted that such publications may not mention a dataset’s citizen science provenance [19]; more consideration should be made on how to better understand and make visible the role citizen science plays in such publications.

Finally, our findings raise the importance of discussion more generally on the role of publication in citizen science. If scientific publication is an important part of the scientific process in general, what role should or must it play in citizen science activities, to distinguish them, for example from political activism or tinkering? Must citizen science activities publish their findings to earn the name and if so, in what form and for what audience? Different formats may be suitable in different contexts in order to make results more accessible for specific audiences.

### The diversity of research focus and scientific disciplines

The disciplinary diversity represented on both platforms is also greater than has been previously acknowledged. Although biology represents a majority of projects on both platforms, projects in the humanities—particularly historical research—represent a new cluster as yet unseen in the scientific literature. Our experience with these communities suggests interesting synergies with biological citizen science [49], confirming the importance of further research into these activities. In particular, it will be important for future studies of citizen science practice to acquaint themselves with publication practices in the humanities, which are not represented in Web of Science, in order to develop better methods to capture activities there.

We find that citizen science is indeed a research approach used in multiple disciplines in all fields of research. At least in Germany and Austria, citizen science can be understood in a cross-disciplinary way. In addition, we found numerous citizen science activities difficult to classify into a single discipline. One example is Project Roadkill, which allows users of a smartphone app to input sightings of roadkill on Austrian roads [47,50]. Although the project was initiated by doctoral students at the Institute of Zoology at the Universität für Bodenkultur Wien, it aims to improve landscape planning decisions in order to protect wildlife [47]. Project Roadkill can thus be understood as interdisciplinary, and could be expected to publish in multiple disciplinary venues. Indeed, the project has already published findings in *Human Computation*, a third disciplinary area [50].

### Related approaches and the limitations of “citizen science”

Our survey addresses projects that identify themselves as citizen science. However, in our work on GEWISS, we ran across numerous additional activities similar enough to potentially fit most practitioners’ understanding of citizen science that themselves use other terminology (see Table 1). Their absence from survey research on the practice of citizen science has led to a bias with the term and can lead to the false conclusion that other actors and research disciplines do not engage non-scientists. We call these “related approaches”: they include participatory research approaches such as public history, participatory health research, and transdisciplinary research (among many others, see also [47]), as well as additional science–society interactions such as science communication, science shops, and open science. Here we argue that the term “citizen science” itself is a limitation to capturing the full range of diversity of science–society interactions that could be described by the term.

**Table 1:** Citizen science and related approaches.

		Fits general characteristics of CS	
		*Yes*	*No*
**Identifies itself as CS**	*Yes*	Citizen science	Expand definition *or* reject
	*No*	Related approach	Not citizen science

Table generated from authors’ experiences defining citizen science in workshops, conferences, and events with the German citizen science community.

Participatory health research is an illustrative example. Some projects on the platform are in the field of health research; Kullenberg and Kasperowski (2016) also found publications that use such approaches using their recursive search algorithm [2]. Participatory health research has parallels to citizen science, such as closer collaboration between scientists and citizens (in this case often patients), but several unique considerations due to the sensitive personal nature of the research topics. Participatory health research has established several networks to discuss current issues and share best practice, such as the German *Netzwerk Partizipative Gesundheitsforschung* (Network for Participatory Health Research, PartNet), founded in 2007, and the International Collaboration For Participatory Research established in 2009 [47]]. With established channels that deal more directly with issues faced by researchers and participants, participatory health researchers have no need for the new mantle of citizen science. At the same time, research on (and funding schemes for) citizen science that omit participatory health research terminology create an artificial gap that falsely suggests far less research in this field than is the case. Methodologies such as ours that analyze citizen science platforms may necessarily omit many participatory health activities.

This and countless other approaches are similar enough to citizen science that they could fit within its definition: engagement of non-scientists in the pursuit of scientific knowledge. However, related approaches have built their own communities and networks in which the understanding of participation and the scientific enterprise is clear. Subsuming these activities under citizen science would be inaccurate and negate decades of definitional work and network-building conducted around other terminologies. Creating a more universal (i.e., discipline-independent) understanding of public engagement in scientific research will require new debates with representatives of these various approaches. It is likely that no single terminology will capture relevant activities across all disciplines. The best case may be that additional network-building efforts create new, broader terms that could be used in future survey research.

## Conclusions

This article joins the body of research studying citizen science as a growing phenomenon. We found far stronger engagement from a wider range of actors than has been acknowledged previously, as well as evidence of more complex roles for “citizens” and “scientists” than are often presented by theoretical definitions. In particular, societal actors such as NGOs and scientific societies, and engaged individuals perform important work worthy of further study. Government agencies and the media are also important supporting players in current citizen science practice. A valuable next step may be to supplement surveys like this one with case study research to develop a better understanding of the different ways these (and other) actors are involved in citizen science.

Our findings also support previous findings that citizen science is best established in biological science, but with a wide range of additional activities. Current practice in Germany and Austria also includes several projects in the humanities, particularly historical disciplines, demonstrating a range of activities not previously captured in survey work or the general literature. Projects in the humanities and those initiated by non-scientific actors are documented outside of Web of Science publications, thus requiring different methods if they are to be captured by further survey research. Citizen science is more diverse and multi-faceted than this quantitative analysis can reveal. Citizen science appears to be at least somewhat established in a range of disciplines and practiced in numerous interdisciplinary projects. In fact, analysis and clustering is difficult because citizen science projects are often inherently interdisciplinary [51]. Future studies may explore the projects themselves in more depth and analyze modes of cooperation between disciplines. Writers attempting to describe citizen science as a general phenomenon should thus be proactive in learning about the different approaches beyond their own field of expertise.

Finally, we argued that the “gap” in certain disciplines can be at least partly explained by the limitations of the term itself and the diversity of related approaches it fails to capture. We discussed the example of participatory health research as emblematic of a range of disciplines with their own terminology and networks for the collaborative research efforts between scientists and non-scientists. Much could be gained for all practitioners by building bridges between these approaches. To do so, they must come together and discuss concepts and practices that may already be settled within their current network. Further studies of citizen science that seek to transcend disciplinary boundaries should be sensitive to the different terminologies used in various disciplines—as that term is only one in a far greater universe of participatory research practice.

## Supporting information

S1 Appendix Source data for platform analysis (N = 97). (XLSX)
